# Impact of alcohol consumption on hyperuricemia and gout: a systematic review and meta-analysis

**DOI:** 10.3389/fnut.2025.1588980

**Published:** 2025-05-20

**Authors:** Weiwei Ma, Guancheng Ye, Yiying Liu, Weikang Sun, Xin Huang, Liekui Hu, Lingting Chen, Binbing Huang, Huanan Li

**Affiliations:** ^1^Jiangxi University of Chinese Medicine, Nanchang, China; ^2^Dongzhimen Hospital, Beijing University of Chinese Medicine, Beijing, China; ^3^Guang’anmen Hospital, China Academy of Chinese Medical Sciences, Beijing, China; ^4^Hospital Affiliated to Jiangxi University of Chinese Medicine, Nanchang, China

**Keywords:** alcohol consumption, gout, hyperuricemia, meta-analysis, systematic evaluation

## Abstract

**Objective:**

With the rapid development of socio-economic conditions, the prevalence of hyperuricemia and gout has steadily increased, significantly impacting individuals’ quality of life. Among various dietary factors, alcohol consumption plays a crucial role in the onset and progression of these conditions. Despite its importance, systematic evaluations of the impact of alcohol consumption on hyperuricemia and gout remain limited. Therefore, this study conducts a meta-analysis to explore these effects, with a focus on the moderating roles of drinking frequency, gender, and other relevant factors.

**Methods:**

A comprehensive literature search was performed using PubMed, Embase, Cochrane, and Web of Science databases up to November 2024. Studies assessing the relationship between alcohol consumption and hyperuricemia or gout were rigorously screened and subjected to quality evaluation. Data extraction and statistical analyses were conducted using STATA 16.0 software. A dose–response analysis was performed to assess the relationship between drinking frequency and disease risk. Regression analysis explored the potential effects of gender, age, country, study type, type of alcohol, and diagnostic criteria on the outcomes.

**Results:**

The meta-analysis revealed that alcohol consumption significantly increases the risk of hyperuricemia and gout (OR = 1.69, 95% CI: 1.47–1.94; Z = 7.494, *p* < 0.05), indicating that drinkers have a 69% higher risk compared to non-drinkers. Egger’s test results (*p* = 0.317, *p* > 0.05) showed no significant publication bias, and sensitivity analyses confirmed the robustness of the findings after excluding individual studies. Dose–response analysis demonstrated a positive association between drinking frequency and the risk of hyperuricemia and gout. Regression analysis indicated that age, country, study type, type of alcohol, and diagnostic criteria had minimal effects on the results, while male were more vulnerable to alcohol-related hyperuricemia and gout than female.

**Conclusion:**

The findings from this meta-analysis confirm that alcohol consumption significantly elevates the risk of hyperuricemia and gout, with higher drinking frequency linked to increased risk. Additionally, male drinkers showed a substantially higher risk compared to female drinkers. These results provide strong evidence supporting the development of public health policies aimed at preventing and controlling hyperuricemia and gout, while offering a foundation for future in-depth research.

**Systematic review registration:**

This study has been registered in the International Prospective Register of Systematic Reviews (PROSPERO). The registration details are as follows: CRD42024361042 (https://www.crd.york.ac.uk/PROSPERO/).

## 1 Introduction

Hyperuricemia and gout are metabolic diseases caused by purine metabolism disorders, forming a continuous pathological process (1). Given their increasing global burden and clinical consequences, it is essential to understand their prevalence and associated health impacts. Over the past 20 years, the global incidence of gout has risen by 63.44%, and years of life lost due to the disease have increased by 51.12% (2). As of 2020, the global prevalence of gout ranges from 1% to 6.8%, with the number of cases projected to exceed 120 million by 2035. This trend makes gout one of the most common forms of inflammatory arthritis in Western countries (3). Hyperuricemia and gout result in elevated serum uric acid levels, which can lead to the deposition of urate crystals in joints, tendons, bursae, and other tissues. Such deposition may trigger acute inflammation and complications, including uric acid kidney stones (4). Furthermore, these conditions often coexist with other metabolic disorders such as metabolic syndrome, chronic kidney disease, diabetes, dyslipidemia, stroke, and cardiovascular diseases. Their presence significantly increases the risk of morbidity and mortality (5). With ongoing economic development and changes in diet and lifestyle, the prevalence of gout and hyperuricemia continues to rise, posing serious public health challenges worldwide. The multiple complications and comorbidities associated with these diseases not only impact individual health and quality of life but also place a significant burden on healthcare systems globally. This underscores the urgent need for targeted public health strategies and early intervention measures to address and manage these conditions effectively (6).

Dietary factors, particularly alcohol consumption, play a crucial role in the onset and progression of hyperuricemia and gout. Among these, alcohol has become one of the most extensively studied risk factors (7). However, the impact of different types of alcoholic beverages—such as beer, spirits, and wine—on the risk of hyperuricemia and gout attacks remains controversial. Some studies suggest that alcohol intake significantly increases the likelihood of gout flare-ups, with men appearing to be more susceptible than females (8–11). On the other hand, other researchers have questioned these findings, arguing that using non-drinkers as a reference group in previous studies may introduce reverse causality, thereby affecting both the strength and direction of the observed associations between alcohol consumption and gout risk (12). To resolve these inconsistencies, this study conducted a systematic review and meta-analysis of relevant literature, specifically examining the effects of alcohol consumption, drinking frequency, and different types of alcoholic beverages on the risk of gout attacks. The results aim to provide evidence-based recommendations for clinical gout management, ultimately supporting the advancement of precision medicine and personalized treatment approaches.

## 2 Materials and methods

### 2.1 Protocol and registration

To ensure methodological rigor and transparency, this meta-analysis followed the MOOSE (Meta-analysis of Observational Studies in Epidemiology) guidelines (13) and the PRISMA (Preferred Reporting Items for Systematic Reviews and Meta-Analyses) declaration (14). These frameworks provide standardized methodologies for conducting and reporting systematic reviews and meta-analyses. Furthermore, to enhance research transparency and minimize the risk of bias, the study protocol was registered with PROSPERO (CRD42024361042), an international database for systematic review protocols.

### 2.2 Literature search strategy

To ensure comprehensive literature coverage, we searched four major databases: PubMed, Embase, Cochrane, and Web of Science, focusing on studies examining the association between alcohol consumption and hyperuricemia or gout from the establishment of these databases to November 2024. Search terms included alcohol-related keywords such as “Alcohol Drinking” “Ethanol” “Alcohol Consumption” “Alcohol Intake” “Beer” “Wine” and “Spirits” as well as disease-related terms like “Hyperuricemia” “Uric Acid” “Gout” “Gouty Arthriti” “Uarthritis” “Arthrolithiasis” “Podagra” and “Metabolic Arthritis.” This systematic search strategy was designed to ensure broad inclusion of relevant literature, thereby enhancing the reliability and comprehensiveness of the study findings.

### 2.3 Inclusion criteria

To evaluate the association between alcohol consumption and hyperuricemia or gout, eligible studies must involve either individuals diagnosed with hyperuricemia or gout, or general populations with clearly measured uric acid levels. Additionally, studies are required to report the association using multivariable-adjusted statistical measures, such as relative risk (RR), odds ratio (OR), hazard ratio (HR), along with 95% confidence intervals (CI). While no restrictions are initially placed on study design, to ensure scientific rigor and comparability, only cohort studies, case–control studies, or cross-sectional studies are considered suitable for inclusion.

### 2.4 Excluded criteria

The exclusion criteria for literature screening were defined across four categories. First, in terms of study populations, studies were excluded if they involved non-hyperuricemia or non-gout individuals, non-human subjects, or patients with known genetic disorders of purine metabolism. Second, regarding methodological considerations, studies with inappropriate designs were excluded, including reviews, case reports, survey analyses, conference abstracts, animal experiments, *in vitro* studies, expert opinions, or those deemed to have low methodological quality. Third, studies with data-related issues were excluded, such as those lacking available data, providing insufficient information for meta-analysis, or missing essential diagnostic indicators (e.g., uric acid levels or gout events). Finally, duplicate publications and studies focusing solely on non-clinically relevant outcomes, such as quality of life, were also considered ineligible for inclusion.

### 2.5 Literature screening and data extraction

Two reviewers (Ma and Ye) independently screened the titles, abstracts, and full texts of the retrieved articles from each database based on predefined eligibility criteria. In case of discrepancies, the original articles were re-evaluated, and disagreements were resolved through discussion to reach consensus. Subsequently, relevant data were extracted from the included studies, including the author, publication year, country, study design, type of alcohol, disease type, diagnostic criteria, sample size, age, and gender. These data were then organized into a standardized three-line table to facilitate systematic analysis of study characteristics.

### 2.6 Literature quality assessment

According to the recommendations of the Agency for Healthcare Research and Quality (AHRQ) for evaluating observational studies, the Newcastle-Ottawa Scale (NOS) was adopted to assess the quality of case–control studies (15). Two reviewers independently evaluated each study using eight specific criteria: appropriateness of case definition, representativeness of cases, selection and definition of controls, comparability between cases and controls, determination of exposure, assessment methods, and non-response rate. Based on the total NOS scores, studies were categorized as low (1–3), moderate (4–6), or high quality (7–9). In addition, each individual item was rated as having low risk, high risk, or unclear risk of bias. Any discrepancies between the reviewers were resolved through discussion to ensure consistent and accurate quality assessments.

### 2.7 Statistical analysis

The extracted data were subjected to meta-analysis using STATA 16.0 software, following a systematic process. First, the odds ratio (OR) was selected as the effect size indicator, and its 95% confidence interval (CI) was calculated to determine statistical significance. Heterogeneity among studies was assessed using the *p*-value and I^2^ statistic. A fixed-effect model (FE) was applied when *p* > 0.1 and I^2^ ≤ 50%, indicating low heterogeneity. In contrast, a random-effects model (RE) was used when *p* ≤ 0.1 and I^2^ > 50%, indicating high heterogeneity (16). To assess publication bias, funnel plots were generated, and Egger’s test was performed to identify potential bias in studies on alcohol consumption and hyperuricemia or gout (17). Sensitivity analysis was conducted using the leave-one-out method to evaluate the robustness of the results and ensure the reliability of the conclusions (18). Finally, meta-regression and subgroup analyses were performed to explore potential sources of heterogeneity and examine their influence on study outcomes, thereby improving the accuracy and scientific validity of the meta-analysis (19).

To determine the dose–response relationship between alcohol consumption and hyperuricemia or gout, methods proposed by Greenland and Longnecker (20) and further developed by Orsini et al. (21) were employed. This approach requires data from at least three quantitative exposure categories, including the total number of participants, person-years, or cases, as well as the corresponding odds ratio (OR) estimates and their variances. For each included study, the median or mean level of alcohol consumption for each category was assigned to the corresponding risk estimate. In instances where these values were not reported, the midpoint between the upper and lower boundaries of each category was used. For open-ended categories (e.g., ≥30 g/day), the width of the adjacent category was used to estimate the midpoint, following the method described by Zhao et al. (22). In cases where the reference group was not the lowest exposure category, relative risk estimates were recalculated using the method proposed by Hamling et al. (23) to ensure consistency across studies. To maintain the integrity of the dose–response analysis, only studies with adequate quantitative information for dose assignment and risk estimation were included. Studies lacking specific data on alcohol intake levels or the number of cases per exposure category were excluded from this portion of the analysis, in line with established research practices (24). To investigate potential non-linear associations between alcohol consumption and the incidence of hyperuricemia or gout, restricted cubic spline modeling was conducted using three knots placed at the 25th, 50th, and 75th percentiles of alcohol intake distribution. The *p*-value for non-linearity was derived by testing the null hypothesis that the coefficient of the second spline was equal to zero.

All statistical analyses were performed using STATA v11.0 software (STATACorp, College Station, TX, United States), with two-sided *p*-values <0.05 considered statistically significant.

## 3 Research results

### 3.1 Results of literature search

**Figure 1:**
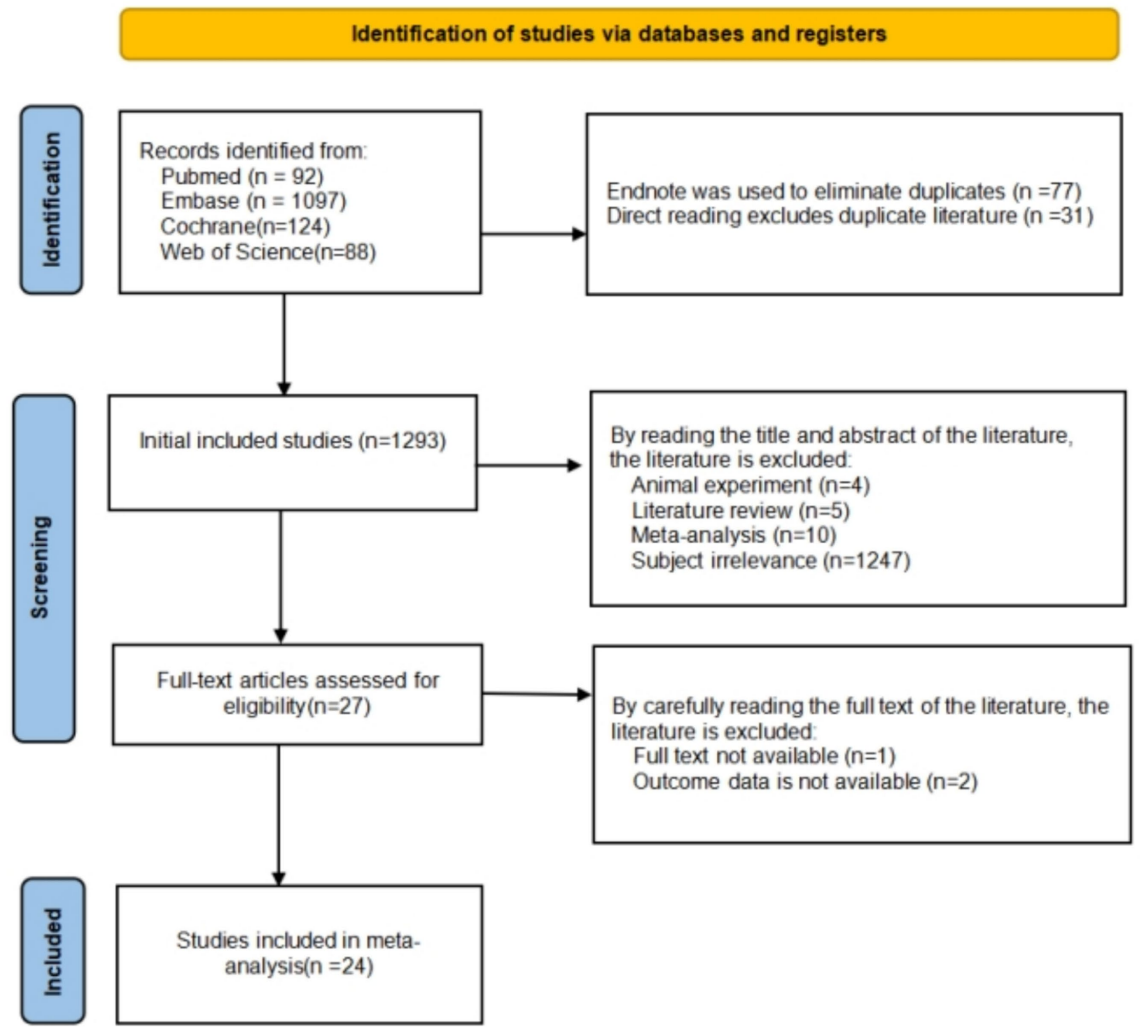
Flowchart of literature selection.

A total of 1,401 articles were initially retrieved. After removing duplicates, 1,293 unique records remained and were systematically screened based on strict eligibility criteria, including study design, participant characteristics, and outcome relevance. After reviewing the titles, abstracts, and full texts, 24 studies were ultimately included in the meta-analysis, providing a robust data foundation for subsequent analyses (as shown in Figure 1).

### 3.2 Results of included study characteristics

The 24 studies included in this research were published between 2003 and 2024, encompassing a total of 1,173,410 participants. Geographically, 7 studies were conducted in China, 6 in the United States, 4 in the United Kingdom, 3 in Japan, 2 in South Korea, 1 in Singapore, and 1 study included populations from both European Caucasians and New Zealand. Regarding study design, 18 were cohort studies and 6 were case–control studies. Among them, 9 studies provided detailed classifications of alcohol types (e.g., beer, spirits, and wine). A total of 11 studies investigated the association between alcohol consumption and laboratory-diagnosed hyperuricemia, while 13 studies focused on clinically diagnosed gout. The age of participants ranged from 20 to 89 years. One study did not report gender information, whereas the remaining 23 studies included 541,655 male and 372,546 female participants. Detailed study characteristics are summarized in Table 1.

**Table 1:** Characteristics of included studies.

Inclusion in the study	Country	Type of study	Type of alcohol	Disease	Diagnostic criteria	Sample size (observation/control group)	Age	Gender (male/female)
Li-Ching Lyu 2003 (48)	China	Case–control Study	Spirits, Wine	Gout	Clinical diagnosis	92/92	20–70	184/0
Hyon K. Choi 2004 (36)	USA	Cohort Study	Beer, Spirits, Wine	Gout	Clinical diagnosis	47,150	54 ± 10	47,150/0
Takuya Sugie 2005 (49)	Japan	Cohort Study	Beer	Hyperuricacidemia	Laboratory examination	715	>35	715/0
Yuqing Zhang 2006 (50)	USA	Cohort Study	Beer, Spirits, Wine	Gout	Clinical diagnosis	197	52 ± 40	158/39
Scott D. Cohen 2008 (51)	USA	Cohort Study	/	Gout	Clinical Diagnosis	259,209	65.36 ± 15.6	/
Paul T. Williams 2008 (52)	USA	Cohort Study	/	Gout	Clinical diagnosis	28,990	44.7 ± 10.9	28,990/0
Vidula Bhole 2010 (53)	USA	Cohort Study	/	Gout	Clinical diagnosis	4,427	46.6 ± 9.0	1,951/2,476
Lucía Cea Soriano 2011 (54)	Britain	Case–control Study	/	Gout	Clinical Diagnosis	24,768/50,000	20–89	54,899/19,869
Seungho Ryu 2011 (55)	Korea	Cohort Study	/	Hyperuricacidemia	Laboratory examination	10,802	37.3 ± 5.0	10,802/0
Hyon K. Choi 2012 (56)	Britain	Case–control Study	/	Gout	Clinical diagnosis	24,768/50.000	20–89	54,899/19,869
K. Nakamura 2012 (57)	Japan	Cohort Study	/	Hyperuricacidemia	Laboratory examination	3,310	38.5 ± 9.60	3,310/0
Chan Hee Lee 2013 (58)	Korea	Case–control Study	/	Gout	Clinical diagnosis	18,123/18,123	55.5 ± 10.2	28,876/7,370
Gim Gee Teng 2013 (59)	Singapore	Cohort Study	Beer, Spirits	Hyperuricacidemia	Laboratory examination	171/312	59.5 ± 8.1	214/269
Humaira Rasheed 2013 (60)	European Caucasians and New Zealand	Case–control Study	Beer, Spirits, Wine	Gout	Clinical Diagnosis	1,431/1,205	51.0 ± 16.0	1,750/886
Yangang Wang 2013 (61)	China	Cohort Study	Beer, Spirits, Wine	Hyperuricacidemia	Laboratory examination	659	47 ± 55.6	436/223
Z. Xiong 2013 (62)	China	Cohort Study	/	Hyperuricacidemia	Laboratory examination	856	60–102	0/856
Chang-Fu Kuo 2014 (63)	Britain	Case–control Study	/	Gout	Clinical diagnosis	39,111/39,111	62.2 ± 15.1	56,700/21,522
Tuhina Neogi 2014 (64)	USA	Cohort Study	/	Gout	Clinical diagnosis	724	54.5 ± 12.5	568/156
Takashi Makinouchi 2016 (65)	Japan	Cohort Study	/	Hyperuricacidemia	Laboratory examination	8,097	42.9 ± 12.0	8,097/0
Zhao Li 2016 (66)	China	Cohort Study	/	Hyperuricacidemia	Laboratory examination	11,039	53.9 ± 10.5	4,997/6,042
Lixian Zhong 2022 (67)	China	Cohort Study	/	Hyperuricacidemia	Laboratory examination	2,031/9,144	55 ± 12.6	5,065/6,110
Xianbin Ding 2023 (68)	China	Cohort Study	/	Hyperuricacidemia	Laboratory examination	22,449	30–79	10,512/11,937
Yueying Wu 2023 (69)	China	Cohort Study	Beer, Wine	Hyperuricacidemia	Laboratory examination	7,083	50.2 ± 15.3	3,418/3,665
Jie-Qiong Lyu 2024 (70)	Britain	Cohort Study	Beer, Spirits, Wine	Gout	Clinical diagnosis	489,221	56 ± 8.1	217,964/271,257

### 3.3 Results of literature quality evaluation

The quality of the 24 included studies was assessed using the Newcastle-Ottawa Scale (NOS), which evaluates methodological rigor based on three domains: selection of study groups, comparability of groups, and ascertainment of outcomes. All studies scored between 8 and 9 points, indicating consistently high methodological quality. These results reflect the strong scientific rigor and reliability of the included studies, providing a robust foundation for the meta-analysis (see Table 2).

**Table 2:** NOS quality assessment.

Included studies	Selection (0–4 points)				Comparability (0–2 points)	Outcome (0–3 points)			Total score
	Appropriateness of case definition	Representativeness of cases	Selection of controls	Definition of controls	Comparability of cases and controls	Exposure assessment	Method of exposure assessment	Non-response Rate	
Li-Ching Lyu 2003 (48)	1	1	1	1	2	1	1	0	8
Hyon K. Choi 2004 (36)	1	1	1	1	2	1	1	1	9
Takuya Sugie 2005 (49)	1	1	1	1	2	1	1	1	9
Yuqing Zhang 2006 (50)	1	1	1	1	2	1	1	0	8
Scott D. Cohen 2008 (51)	1	1	1	1	2	1	1	1	9
Paul T Williams 2008 (52)	1	1	1	1	2	1	1	1	9
Vidula Bhole 2010 (53)	1	1	1	1	2	1	1	1	9
Lucía Cea Soriano 2011 (54)	1	1	1	1	2	1	1	0	8
Seungho Ryu 2011 (55)	1	1	1	1	2	1	1	1	9
Hyon K. Choi 2012 (56)	1	1	1	1	2	1	1	0	8
K. Nakamura 2012 (57)	1	1	1	1	2	1	1	1	9
Chan Hee Lee 2013 (58)	1	1	1	1	2	1	1	0	8
Gim Gee Teng 2013 (59)	1	1	1	1	2	1	1	0	8
Humaira Rasheed 2013 (60)	1	1	1	1	2	1	1	1	9
Yangang Wang 2013 (61)	1	1	1	1	2	1	1	0	8
Z. Xiong 2013 (62)	1	1	1	1	2	1	1	1	9
Chang-Fu Kuo 2014 (63)	1	1	1	1	2	1	1	0	8
Tuhina Neogi 2014 (64)	1	1	1	1	2	1	1	0	8
Takashi Makinouchi 2016 (65)	1	1	1	1	2	1	1	1	9
Zhao Li 2016 (66)	1	1	1	1	2	1	1	1	9
Lixian Zhong 2022 (67)	1	1	1	1	2	1	1	1	9
Xianbin Ding 2023 (68)	1	1	1	1	2	1	1	1	9
Yueying Wu 2023 (69)	1	1	1	1	2	1	1	1	9
Jie-Qiong Lyu 2024 (70)	1	1	1	1	2	1	1	1	9

### 3.4 Results of meta-analysis

#### 3.4.1 Relationship between alcohol consumption and hyperuricacidemia/gout

**Figure 2:**
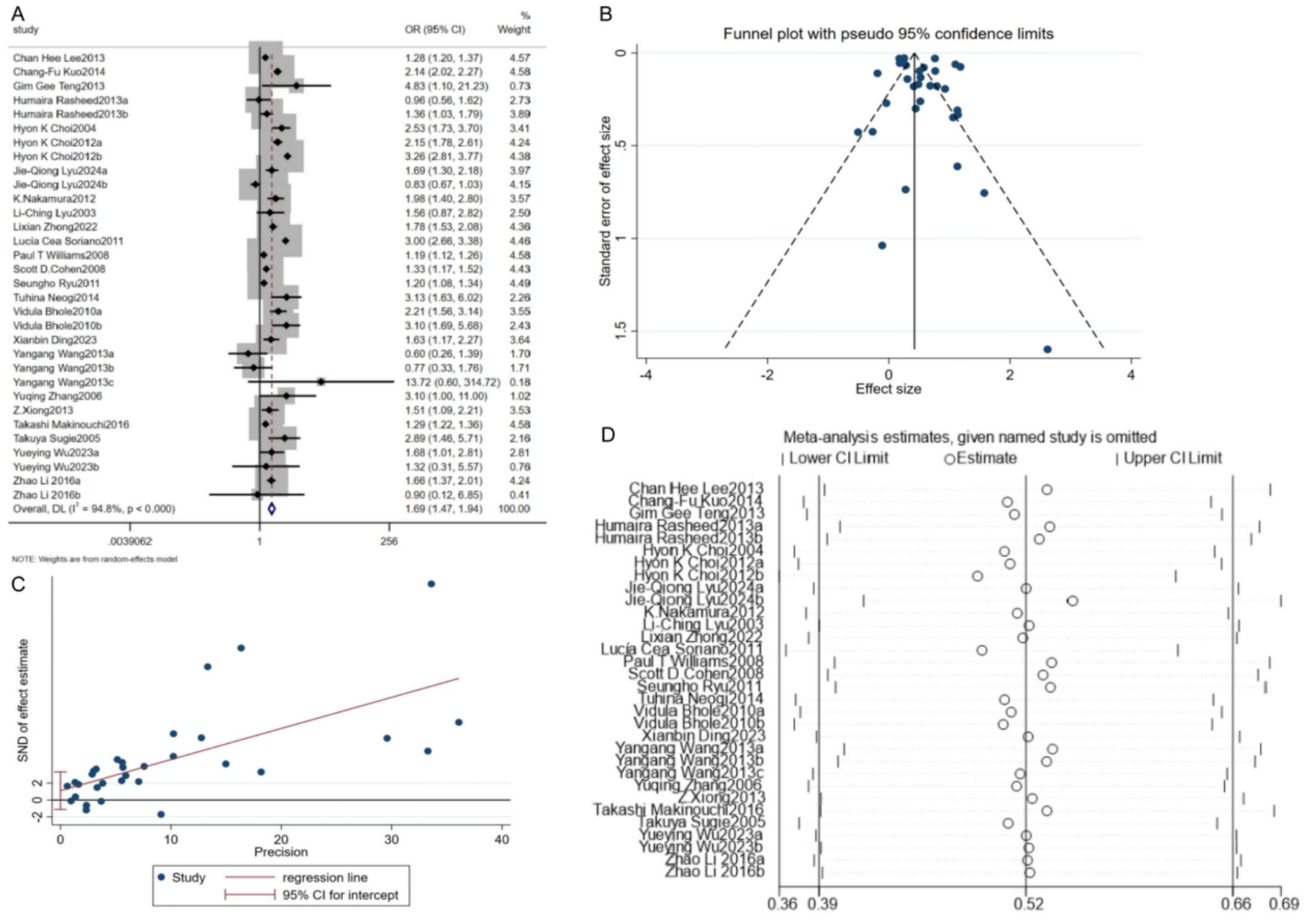
Relationship between alcohol consumption and hyperuricacidemia/gout. **(A)** Forest plot; **(B)** Funnel plot; **(C)** Egger’s test; **(D)** Sensitivity analysis.

This meta-analysis included 24 studies examining the association between alcohol consumption and hyperuricemia or gout. Heterogeneity testing revealed significant variability among studies (*p* < 0.05, I^2^ = 94.8%), warranting the use of a random-effects model for pooled effect estimation. The results showed that alcohol consumption was significantly associated with an increased risk of hyperuricemia or gout, with a combined OR of 1.69 (95% CI: 1.47–1.94; Z = 7.494, *p* < 0.05), indicating a 69% higher risk in alcohol consumers compared to non-drinkers (Figure 2A). Publication bias was evaluated using a funnel plot and Egger’s test. The scatter points in the funnel plot were symmetrically distributed, with only a few studies falling outside the 95% confidence limits. Egger’s test yielded a *p*-value of 0.317 (*p* > 0.05), suggesting no significant publication bias (Figures 2B,C). Furthermore, sensitivity analysis was performed using the leave-one-out approach. The pooled OR remained stable throughout, indicating that the overall results were robust and reliable (Figure 2D).

**Figure 3:**
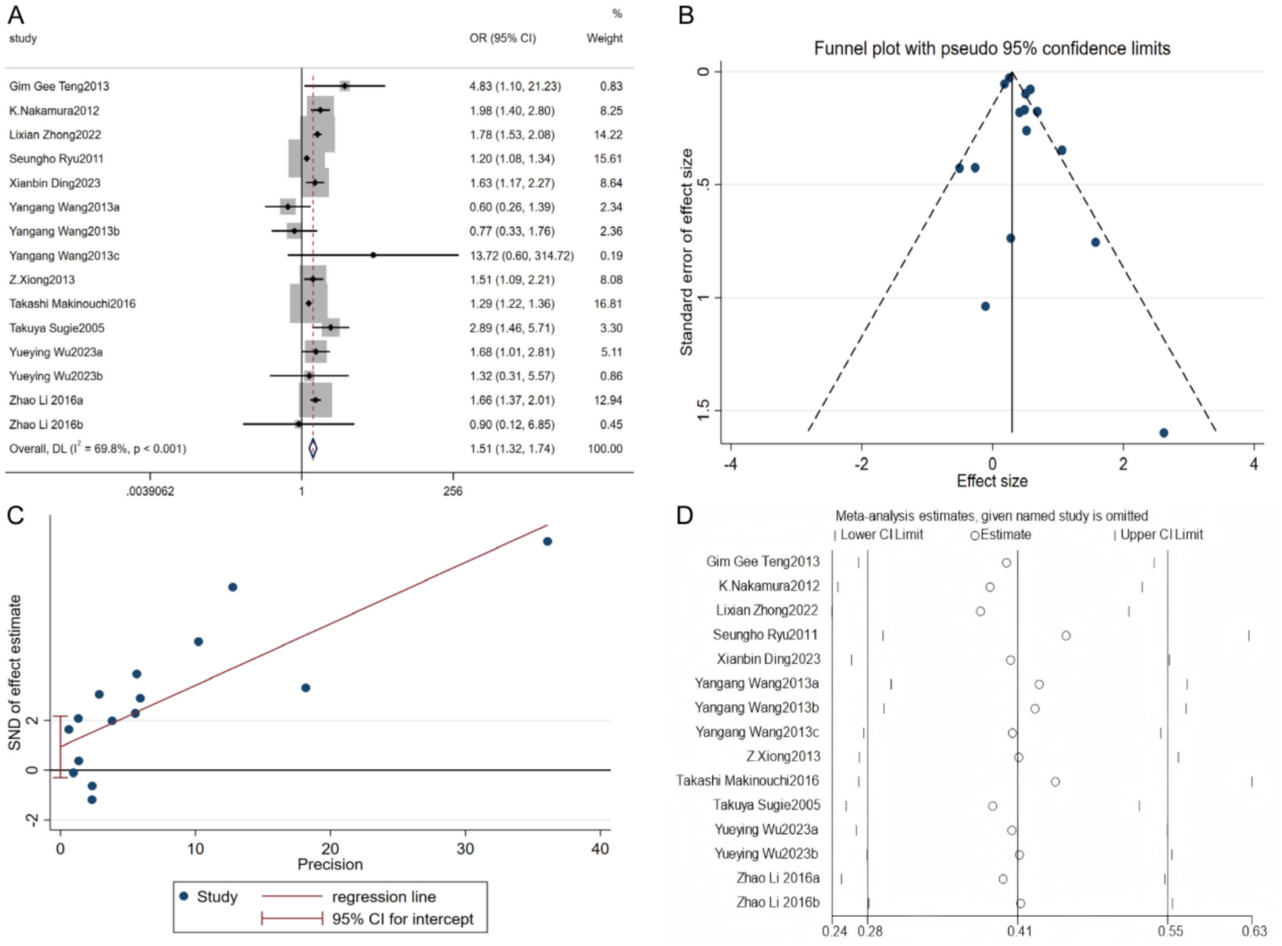
Relationship between alcohol consumption and hyperuricacidemia. **(A)** Forest plot; **(B)** Funnel plot; **(C)** Egger’s test; **(D)** Sensitivity analysis.

Eleven studies investigated the association between alcohol consumption and hyperuricemia. The heterogeneity test showed significant heterogeneity (*p* < 0.001, I^2^ = 69.8%), so a random-effects model was used for effect size aggregation analysis. The meta-analysis results indicated that alcohol consumption significantly increases the risk of hyperuricemia [OR = 1.51, 95% CI (1.32, 1.74), Z = 5.886, *p* < 0.001] (Figure 3A). Regarding publication bias, the funnel plot showed slight asymmetry with more scattered points on the right side, and some studies were located outside the lower boundary. However, the Egger test (*p* = 0.123) did not reach statistical significance, indicating no significant publication bias risk was observed (Figures 3B,C). Sensitivity analysis showed that after excluding each study one by one, the pooled effect size did not change significantly, suggesting that the results are robust and reliable (Figure 3D).

**Figure 4:**
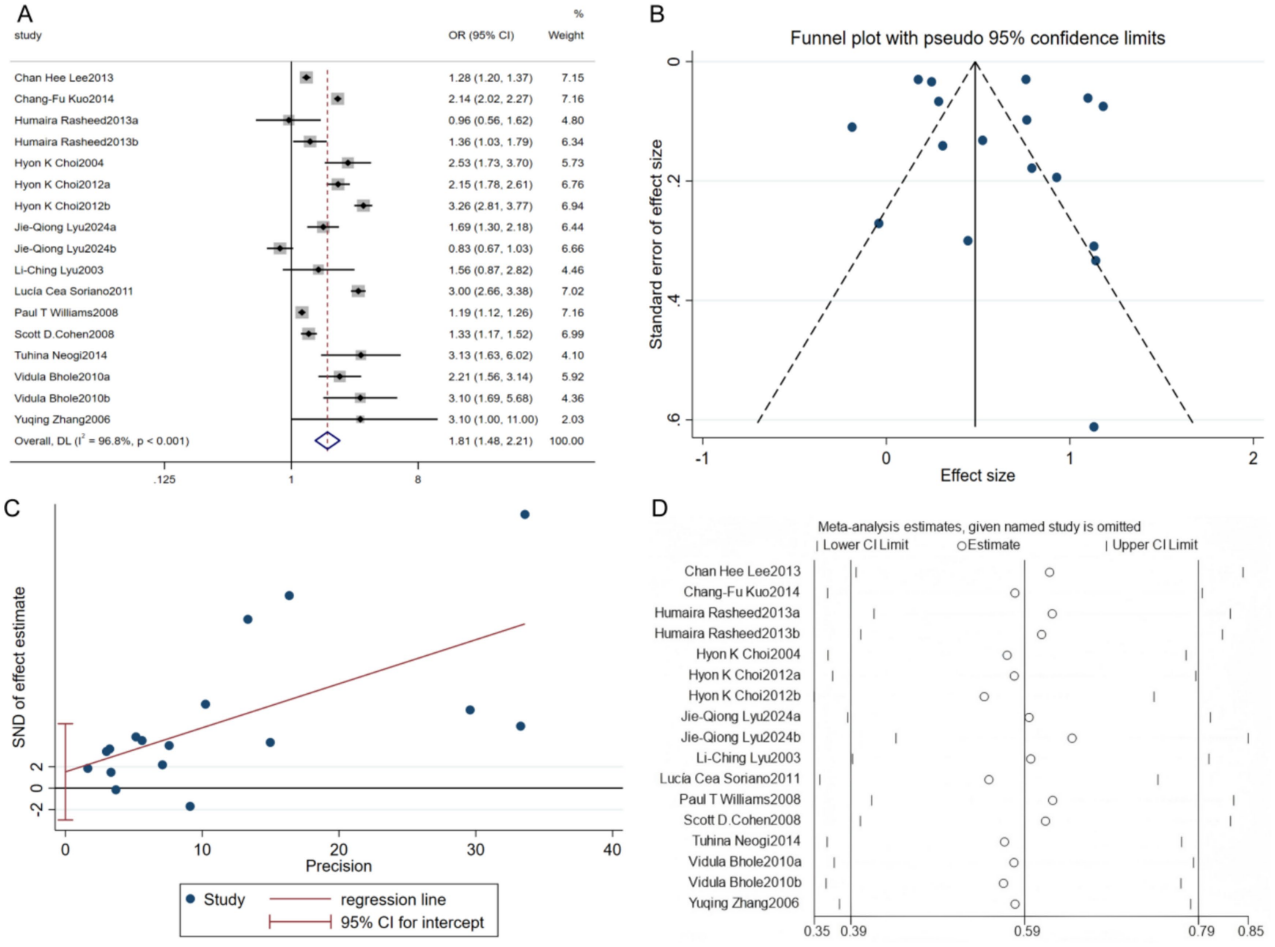
Relationship between alcohol consumption and gout. **(A)** Forest plot; **(B)** Funnel plot; **(C)** Egger’s test; **(D)** Sensitivity analysis.

Twelve studies analyzed the relationship between alcohol consumption and gout. The heterogeneity analysis showed significant differences (*p* < 0.001, I^2^ = 96.8%), so a random-effects model was used for the pooled analysis. The results showed that alcohol consumption significantly increases the risk of gout [OR = 1.81, 95% CI (1.48, 2.21), Z = 5.777, *p* < 0.001] (Figure 4A). The funnel plot showed that the study scatter was generally symmetric, with only a few studies outside the confidence interval. The Egger test result (*p* = 0.481) indicated no significant publication bias (Figures 4B,C). Sensitivity analysis results were stable, and excluding each study one by one showed minimal changes in the effect size, indicating that the meta-analysis conclusion has good robustness and credibility (Figure 4D).

**Figure 5:**
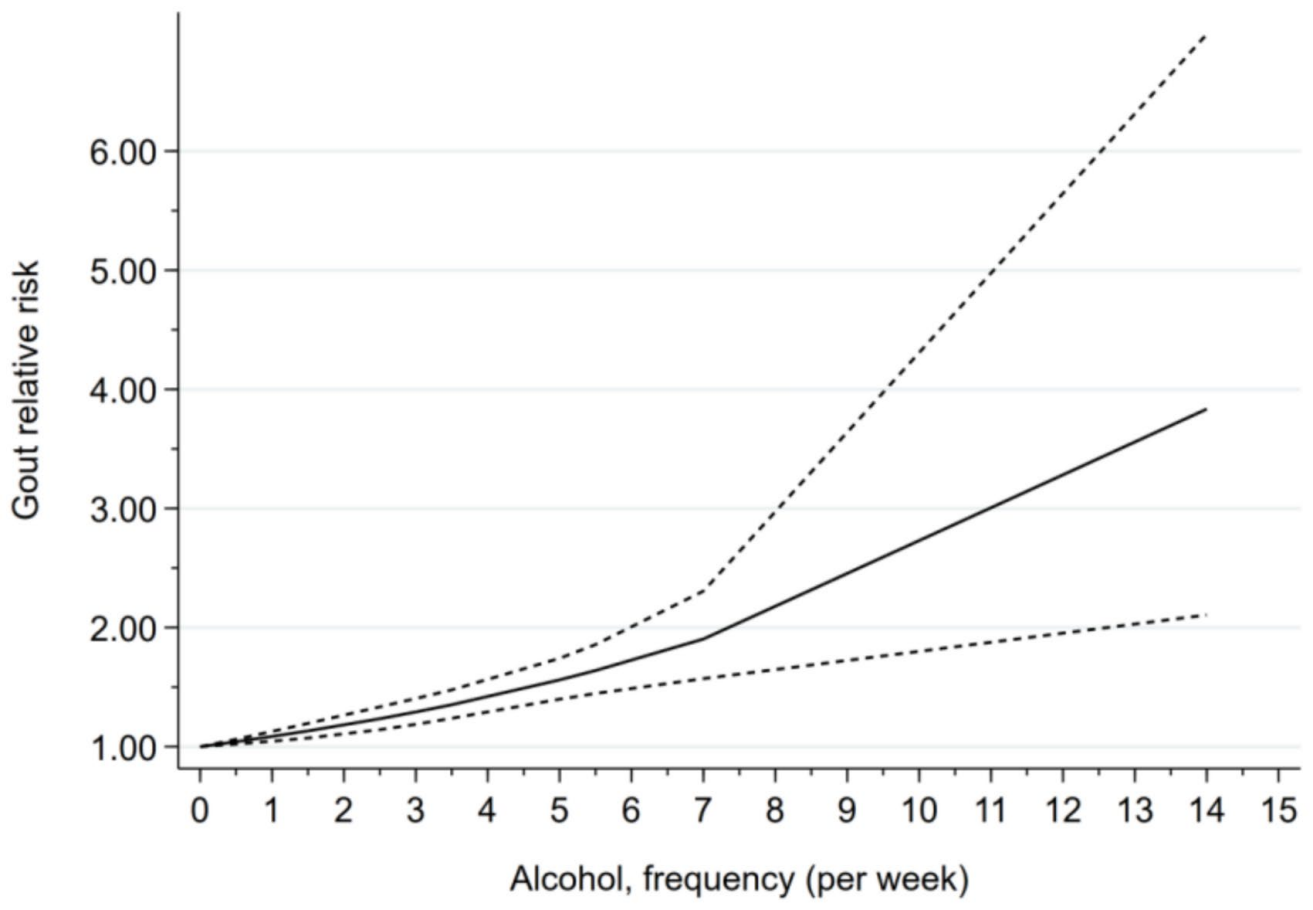
Dose-response analysis of the relationship between alcohol consumption frequency and hyperuricacidemia/gout.

For the nonlinear dose–response analysis, at least three exposure categories were required; thus, studies that only provided continuous estimates or lacked case and participant numbers per category were excluded, resulting in 10 eligible studies. No evidence of a nonlinear association was observed between alcohol consumption frequency and the risk of hyperuricemia or gout (*p* for nonlinearity = 0.6979). Therefore, a restricted cubic spline model was applied to assess the linear dose–response relationship. The analysis revealed a significant positive linear association, indicating that higher frequencies of alcohol consumption were consistently associated with an increased risk of hyperuricemia or gout (Figure 5).

#### 3.4.2 Subgroups

**Figure 6:**
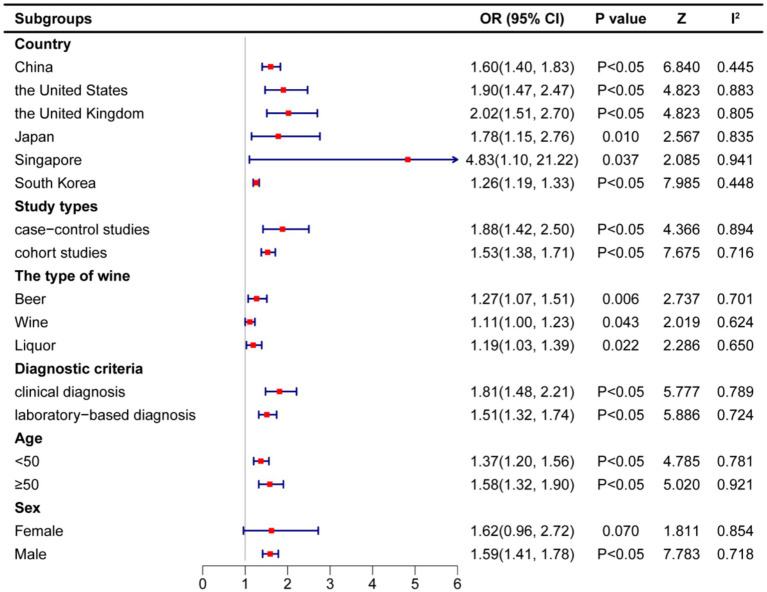
Subgroup analysis of the relationship between alcohol consumption and hyperuricacidemia/gout—forest plot.

To comprehensively assess the potential association between alcohol consumption and hyperuricemia/gout, a subgroup analysis was conducted based on different countries, study types, alcohol types, diagnostic criteria, age, and gender. This analysis helps identify potential influences of various variables and sources of heterogeneity in the study results. The results are shown in Figure 6. The following sections provide a detailed discussion based on these subgroup factors. First, at the country level, significant heterogeneity differences were observed among different countries. South Korea (I^2^ = 0.0%) and China (I^2^ = 18.2%) exhibited low heterogeneity, while America (I^2^ = 86.7%), Japan (I^2^ = 81.7%), Singapore (I^2^ = 100.0%), and Britain (I^2^ = 96.3%) showed high heterogeneity, prompting the use of a random-effects model. Significant associations between alcohol consumption and hyperuricemia/gout were observed across all countries. The specific results are as follows: South Korea (OR = 1.26, 95% CI: 1.05–1.51, Z = 7.958, *p* < 0.05); Britain (OR = 1.90, 95% CI: 1.55–2.34, Z = 4.823, *p* < 0.05); Japan (OR = 1.78, 95% CI: 1.44–2.20, Z = 2.567, *p* = 0.01); Singapore (OR = 4.83, 95% CI: 1.10–21.22,Z = 2.085, *p* = 0.037); China (OR = 1.60, 95% CI: 1.40–1.83, Z = 6.840, *p* < 0.05), as shown in Supplementary Figure 1. These findings suggest that geographic differences significantly influence the observed associations. Given the country-specific differences, the study further explored heterogeneity by examining different study types. Both case–control studies (I^2^ = 97.4%) and cohort studies (I^2^ = 80.1%) exhibited high heterogeneity, potentially due to differences in study design, sample selection, and follow-up duration. Nevertheless, alcohol consumption significantly increased the risk of hyperuricemia/gout in both case–control studies (OR = 1.88, 95%CI: 1.42–2.50, Z = 4.366, *p* < 0.05) and cohort studies (OR = 1.53, 95%CI: 1.38–1.71, Z = 7.675, *p* < 0.05), as detailed in Supplementary Figure 2. Study design differences may interact with specific alcohol types, prompting further exploration. Regarding alcohol types, beer (OR = 1.27, 95%CI: 1.07–1.51, Z = 2.737, *p* = 0.006, I^2^ = 70.1%), spirits (OR = 1.19, 95%CI: 1.03–1.39, Z = 2.286, *p* = 0.022, I^2^ = 65%), and wine (OR = 1.11, 95%CI: 1.00–1.23, Z = 2.019, *p* = 0.043, I^2^ = 62.4%) all showed significant associations, albeit with varying effect sizes, as shown in Supplementary Figure 3. Additionally, the analysis explored different diagnostic criteria. Both clinical diagnosis (I^2^ = 96.8%) and laboratory-based diagnosis (I^2^ = 69.8%) demonstrated significant associations between alcohol consumption and hyperuricemia/gout, with OR = 1.81 (95% CI: 1.48–2.21, Z = 5.777, *p* < 0.05) and OR = 1.51 (95% CI: 1.32–1.74, Z = 5.886, *p* < 0.05), respectively, as shown in Supplementary Figure 4. Given that diagnosis can be influenced by patient age, age-specific analyses were conducted. In terms of age differences, the effects of alcohol consumption varied across age groups. Significant associations were observed among individuals with a mean age of <50 years (OR = 1.37, 95% CI: 1.20–1.56, I^2^ = 78.1%, Z = 4.785, *p* < 0.05) and ≥50 years (OR = 1.58, 95% CI: 1.32–1.90, I^2^ = 92.1%, Z = 5.02, *p* < 0.05), with older age linked to a higher risk, as shown in Supplementary Figure 5. Given potential metabolic and hormonal differences by gender, a gender-specific analysis followed. Finally, concerning gender, alcohol consumption was significantly associated with hyperuricemia/gout in males (OR = 1.59, 95% CI: 1.41–1.78, I^2^ = 83.2%, Z = 7.783, *p* < 0.05), while the association in females (OR = 1.62, 95% CI: 0.96–2.72, I^2^ = 77.3%, Z = 1.811, *p* = 0.07) did not reach statistical significance. This suggests that gender differences may influence the alcohol-disease relationship, with men being particularly at risk, as detailed in Supplementary Figure 6.

## 4 Discussion

This systematic review and meta-analysis aimed to systematically assess the association between alcohol consumption and the risk of hyperuricemia and gout, including 24 studies that met the inclusion criteria. The pooled analysis revealed a statistically significant positive correlation between alcohol consumption and the risk of developing hyperuricemia and gout. Further dose–response analysis indicated a nonlinear relationship between alcohol intake and the risk of these diseases, suggesting that as drinking frequency and intake increase, the disease risk also tends to rise, implying a potential cumulative promotive effect of alcohol consumption in disease onset. To assess the robustness of this association and its applicability across different populations, subgroup analyses were conducted. The results showed no significant differences in the observed associations across study regions, study design types, types of alcohol consumed, age groups of participants, and diagnostic criteria for hyperuricemia/gout, indicating a certain degree of generalizability of the findings. However, the gender subgroup analysis revealed that the increased risk was primarily observed in the male population, and the effect of alcohol consumption on hyperuricemia and gout risk in females did not reach statistical significance. Based on the above findings, this study provides reliable evidence for formulating dietary intervention strategies, optimizing nutritional guidelines, and advancing preventive efforts for hyperuricemia and gout.

In recent years, with continuous improvements in living standards and changes in lifestyle, hyperuricemia and gout have gradually become common diseases, closely related to factors such as age, gender, and lifestyle (25). Among these, hyperuricemia is widely considered the pathological basis of gout. Elevated levels of serum uric acid can result in the formation and deposition of monosodium urate crystals in joints and soft tissues, ultimately triggering acute inflammatory responses and joint pain characteristic of gout (26). Although approximately 85–90% of patients with hyperuricemia do not exhibit overt symptoms or experience gout attacks (27), clinical imaging studies have demonstrated that about 30–50% of asymptomatic hyperuricemic individuals show urate crystal deposits in joints or tendons, suggesting a potential predisposition to gout attacks in this population (28, 29). Against this background, the findings of the present study further confirm a significant positive correlation between alcohol consumption and the risk of hyperuricemia and gout, consistent with previous research (30). To further explore the role of alcohol consumption in the pathogenesis of hyperuricemia and gout, several studies have proposed physiological and pathological hypotheses. Firstly, alcoholic beverages—especially beer and spirits—contain high concentrations of purines, whose metabolic byproducts can markedly enhance uric acid production in the body (31). Secondly, lactic acid produced during alcohol metabolism may inhibit the renal excretion of uric acid, leading to elevated serum uric acid levels (32). In addition, accumulating evidence suggests that alcohol consumption may indirectly aggravate uric acid metabolic imbalance by disrupting the gut microbiota (33). Dysbiosis of the gut flora can affect both the synthesis and excretion of uric acid, thereby increasing the risk of hyperuricemia and gout (33). Finally, alcohol may interfere with the kidney’s regulatory mechanisms for uric acid, affecting its filtration, reabsorption, and secretion in the renal tubules, thus further disrupting uric acid homeostasis in the body (34).

Subgroup analysis revealed a significant gender difference in the impact of alcohol consumption on the risk of hyperuricemia and gout, with males being more susceptible to alcohol-related metabolic risks than females. This finding is largely consistent with previous research. One study indicated that hypertension and alcohol consumption are the main risk factors for hyperuricemia and gout in men, whereas smoking shows a stronger risk association among women (35). These findings suggest that gender may play a moderating role in the causal pathway between alcohol intake and hyperuricemia/gout. The potential mechanisms underlying this gender difference may involve both physiological foundations and behavioral patterns. In terms of biological mechanisms, differences in sex hormone levels may play a crucial role. Studies have shown that estrogen can enhance the renal excretion of uric acid, thereby reducing serum uric acid levels and lowering the risk of gout in women (36, 37). However, in postmenopausal women, the abrupt decline in estrogen levels may lead to elevated serum uric acid concentrations, consequently increasing the likelihood of gout onset (38). Moreover, baseline differences in serum uric acid levels between sexes should not be overlooked, as men generally exhibit higher serum uric acid concentrations and are thus more sensitive to alcohol-induced metabolic disturbances (35). From a behavioral perspective, gender differences in drinking patterns may further amplify the impact of alcohol on disease risk. Relevant studies have demonstrated that alcohol intake is significantly higher in men than in women, particularly in terms of the frequency and quantity of beer and spirits consumption, both of which are closely associated with increased serum uric acid levels. In contrast, women are more inclined to consume wine, which has a relatively smaller impact on uric acid metabolism (39). These differences help explain the heterogeneous effects of alcohol on the risk of hyperuricemia and gout across genders.

This study further found that different types of alcoholic beverages exert significantly different effects on the risk of hyperuricemia and gout, with beer associated with the highest risk, followed by spirits, while wine showed relatively lower impact. This conclusion is consistent with findings from previous studies (40). Notably, the influence of beer on hyperuricemia and gout may operate through multiple mechanisms. On one hand, beer is rich in D-amino acids, which can be metabolized into uric acid through specific pathways in the body, thereby increasing endogenous uric acid production (41). On the other hand, ethanol in beer may inhibit the function of urate transporters in the kidney, reducing the efficiency of uric acid excretion and leading to uric acid retention (42). This dual mechanism of “increased production and decreased excretion” may be the primary reason why beer significantly elevates disease risk. Although spirits contain lower levels of purines compared to beer, their high ethanol concentration can promote lactic acid production in the body, which in turn competitively inhibits the renal tubular excretion of uric acid, resulting in elevated serum uric acid levels (43). Therefore, despite their relatively low purine content, spirits still pose a potential risk for inducing hyperuricemia. In contrast, wine appears to have a more complex influence on uric acid metabolism. Although wine (especially red wine) contains ethanol, it is also rich in polyphenolic compounds, which are believed to exert protective effects on uric acid metabolism by reducing oxidative stress and inhibiting xanthine oxidase activity (44). However, this potential benefit is dose-dependent—if the intake exceeds a certain threshold, the harmful effects of ethanol may prevail and offset any protective benefits.

It is noteworthy that the positive correlation between alcohol consumption and the risk of hyperuricemia and gout is more pronounced in individuals over the age of 50, a phenomenon likely influenced by multiple age-related factors. Firstly, as age increases, renal function gradually declines, leading to reduced uric acid excretion and consequently elevated serum uric acid levels. Relevant studies have shown that decreased glomerular filtration rate (GFR) in the elderly is one of the key physiological contributors to the increased prevalence of hyperuricemia (45). Secondly, purine metabolism pathways also undergo age-related alterations, particularly with increased expression and activity of xanthine oxidase (XO) in older populations. XO, a rate-limiting enzyme in purine metabolism, catalyzes the conversion of hypoxanthine to xanthine and subsequently to uric acid; its elevated activity can significantly enhance uric acid production (46). Some studies suggest that this increased enzyme activity may be associated with dysregulated expression across multiple tissues. In addition, lifestyle changes among the elderly may further aggravate uric acid metabolic imbalance. For instance, reduced physical activity and increased intake of purine-rich foods are behavioral factors that can lead to increased uric acid production and impaired excretion (47). Against this backdrop, the metabolic burden induced by alcohol consumption further exacerbates hyperuricemic states, significantly increasing the risk of gout development. In summary, declining renal function, abnormal purine metabolism, and lifestyle changes synergistically amplify the pathogenic impact of alcohol consumption on hyperuricemia and gout during older age. Therefore, special attention should be given to alcohol consumption in individuals over 50 years old to reduce the risk of related metabolic disorders.

Despite employing a meta-analytic approach to systematically synthesize existing literature—thus enhancing statistical power and the robustness of results—this study has certain limitations that warrant attention. Firstly, substantial heterogeneity was observed, which may stem from differences across the included studies, such as variations in dietary patterns among populations, the alcohol content and composition of different alcoholic beverages, and participants’ lifestyles and drinking habits. These factors may lead to considerable variability in effect size estimates, thereby reducing the consistency and credibility of the study’s conclusions. Secondly, publication bias is a common issue in systematic reviews and meta-analyses. Studies with statistically significant findings are more likely to be published, whereas negative or non-significant results are often underreported, which may lead to a systematic overestimation of the effect of alcohol consumption on hyperuricemia or gout and compromise the objectivity of the findings. In addition, most of the included studies were observational in nature and are inherently susceptible to selection bias (e.g., inappropriate participant recruitment), information bias (e.g., self-reported alcohol intake), and residual confounding (e.g., insufficient control of variables such as body weight or medication use), which limit the accuracy of causal inference between alcohol consumption and hyperuricemia/gout. Furthermore, the meta-analysis results may be constrained by the timing of the literature search and publication lag, making it difficult to capture the latest research developments—especially in a rapidly evolving field—thereby diminishing the timeliness and practical applicability of the findings. Therefore, although this study provides valuable evidence-based insights into the association between alcohol consumption and hyperuricemia/gout, practical application of its findings should be guided by results from up-to-date, high-quality prospective cohort studies or randomized controlled trials to further verify and refine the conclusions, thereby enhancing their scientific validity and generalizability.

In summary, alcohol consumption is closely associated with the risk of gout and hyperuricemia, and this risk increases with the frequency and amount of alcohol consumed, particularly in the male population, where the association is more pronounced. The dose–response analysis in this study further confirms that even low levels of alcohol consumption can adversely affect disease risk, while high levels of drinking significantly exacerbate this risk. Therefore, reasonable control of drinking behavior, particularly limiting excessive alcohol intake in high-risk populations (such as middle-aged and elderly men), is of great importance for the prevention and management of gout and hyperuricemia. In clinical practice, a comprehensive approach considering individual drinking habits, gender, age, and comorbidities should be adopted to develop targeted health interventions, such as controlling alcohol consumption frequency, adjusting the types of alcoholic beverages consumed, and enhancing lifestyle management. Future research could further focus on the biological mechanisms through which alcohol triggers metabolic disturbances, including the regulation of xanthine oxidase activity, renal uric acid excretion pathways, gut microbiota dysbiosis, and systemic inflammatory responses, with the aim of providing a stronger theoretical foundation and practical guidance for the precise prevention and intervention of hyperuricemia and gout.

## Data Availability

The original contributions presented in the study are included in the article/Supplementary material, further inquiries can be directed to the corresponding author.
