# Supplementary material for: Impact of alcohol consumption on hyperuricemia and gout: a systematic review and meta-analysis
Source: Front Nutr. 2025 May 20;12:1588980. doi: 10.3389/fnut.2025.1588980 (PMC12129753; doi:10.3389/fnut.2025.1588980)
Supplement: Supplementary file 1 [file Image_1.pdf]

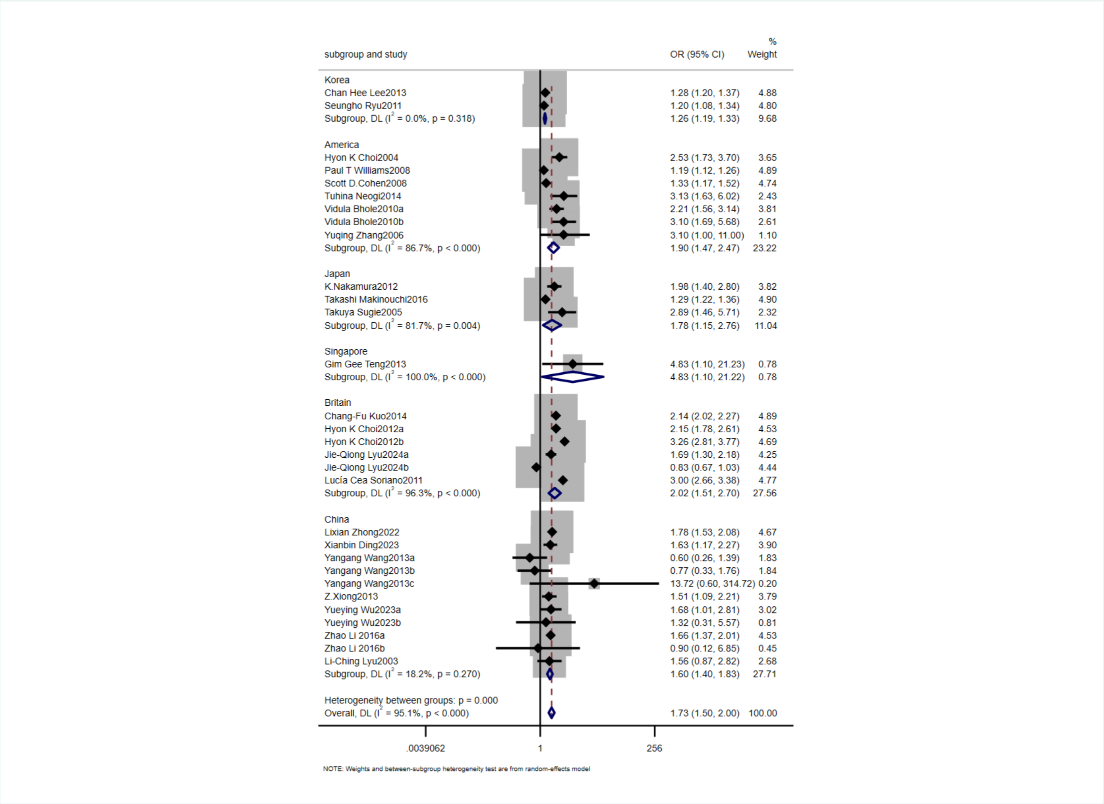

Supplementary Figure 1. Subgroup analysis of the relationship between alcohol consumption and hyperuricacidemia/gout by country- Forest Plot.

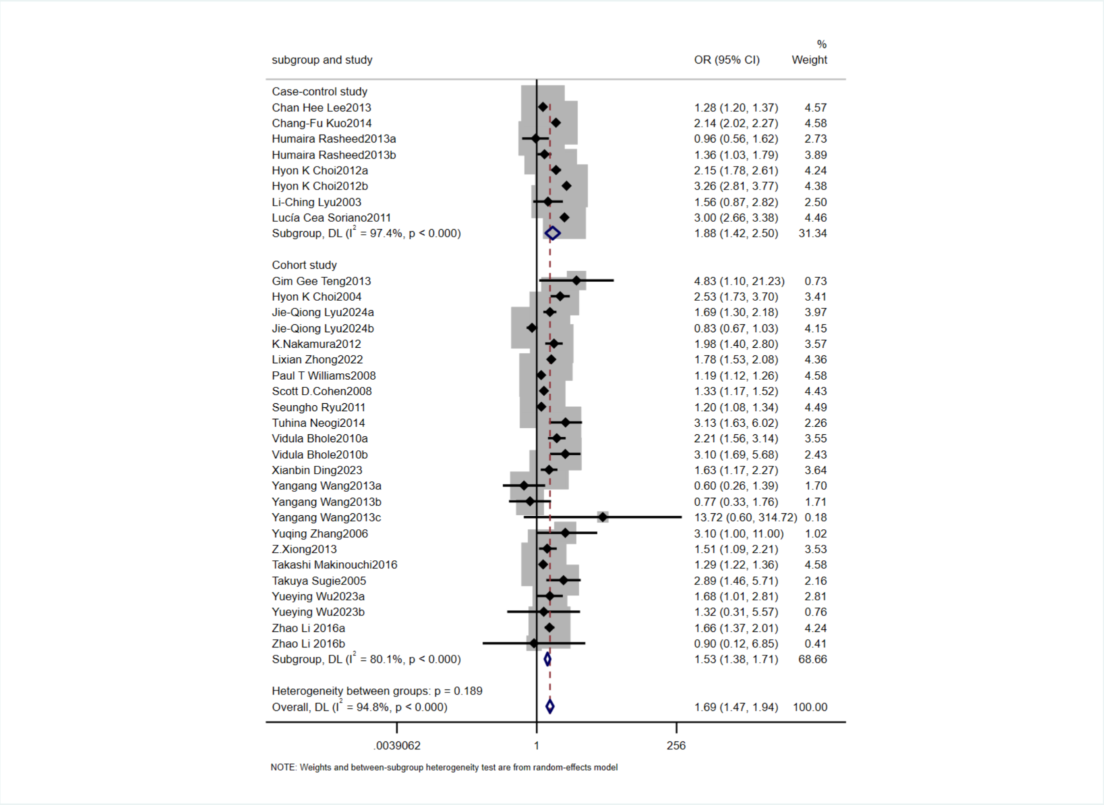

Supplementary Figure 2. Subgroup analysis of the relationship between alcohol consumption and hyperuricacidemia/gout by study type- Forest Plot.

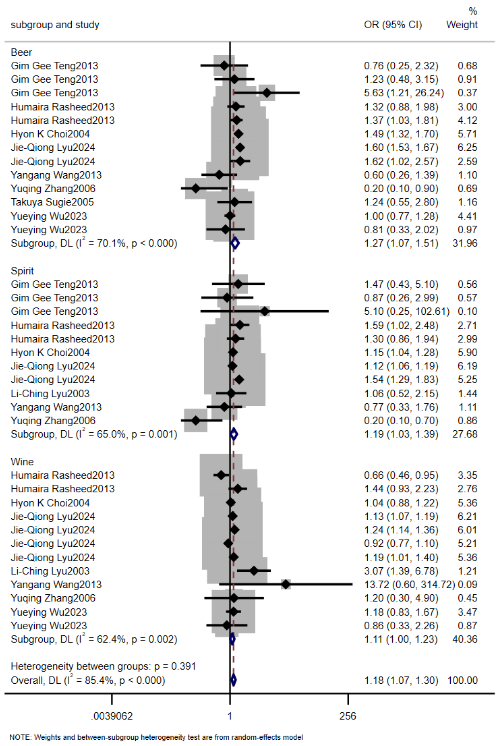

Supplementary Figure 3. Subgroup analysis of the relationship between alcohol consumption and hyperuricacidemia/gout by diagnostic criteria- Forest Plot.

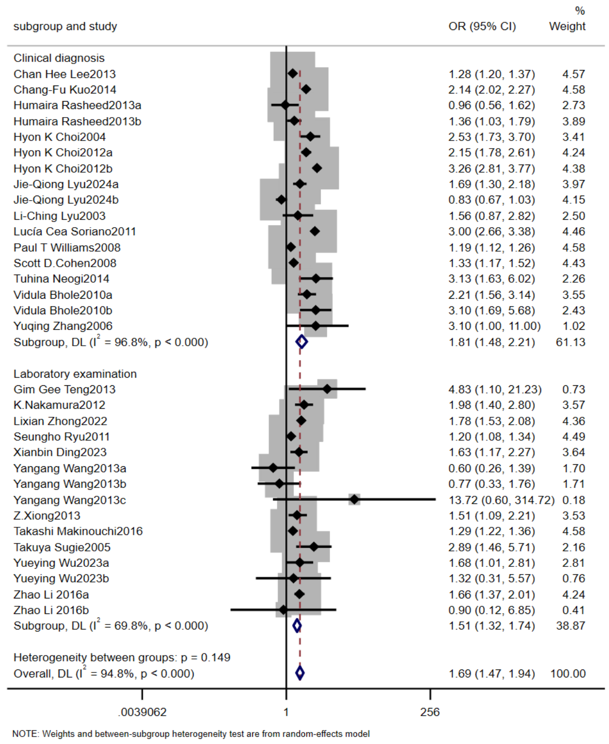

Supplementary Figure 4. Subgroup analysis of the relationship between alcohol consumption and hyperuricacidemia/gout by type of alcohol- Forest Plot.

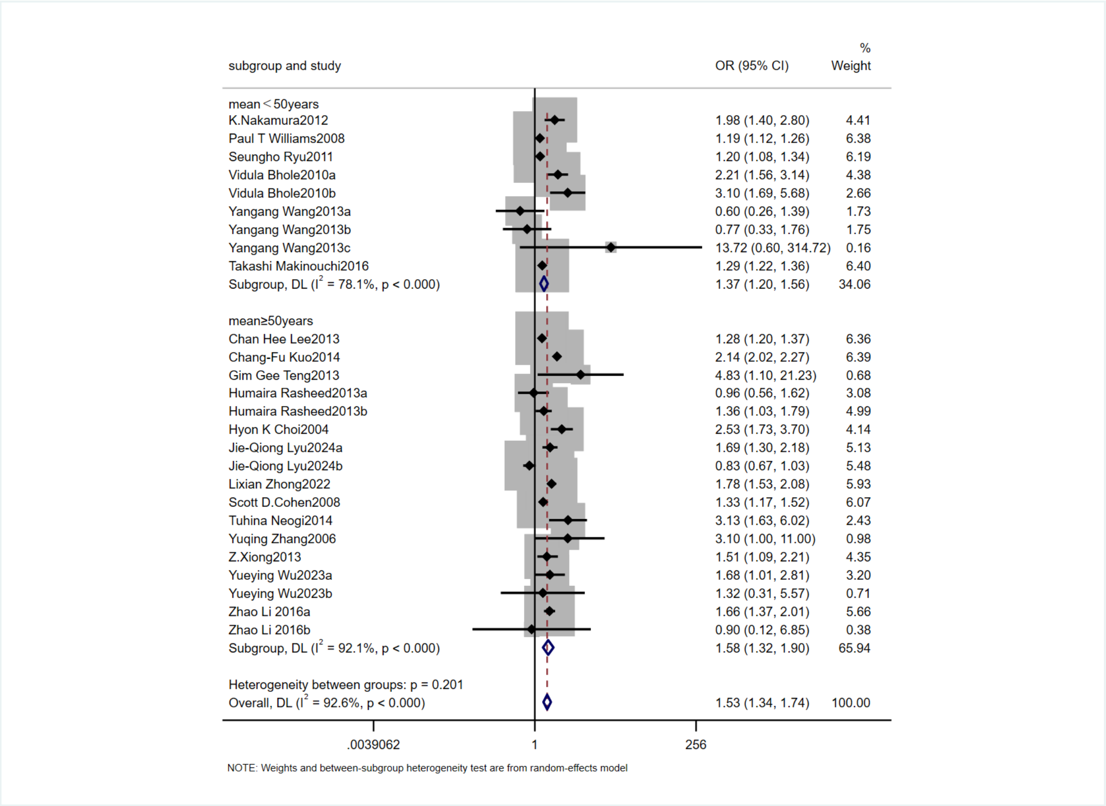

Supplementary Figure 5. Subgroup analysis of the relationship between alcohol.consumption and hyperuricacidemia/gout by age - Forest Plot.

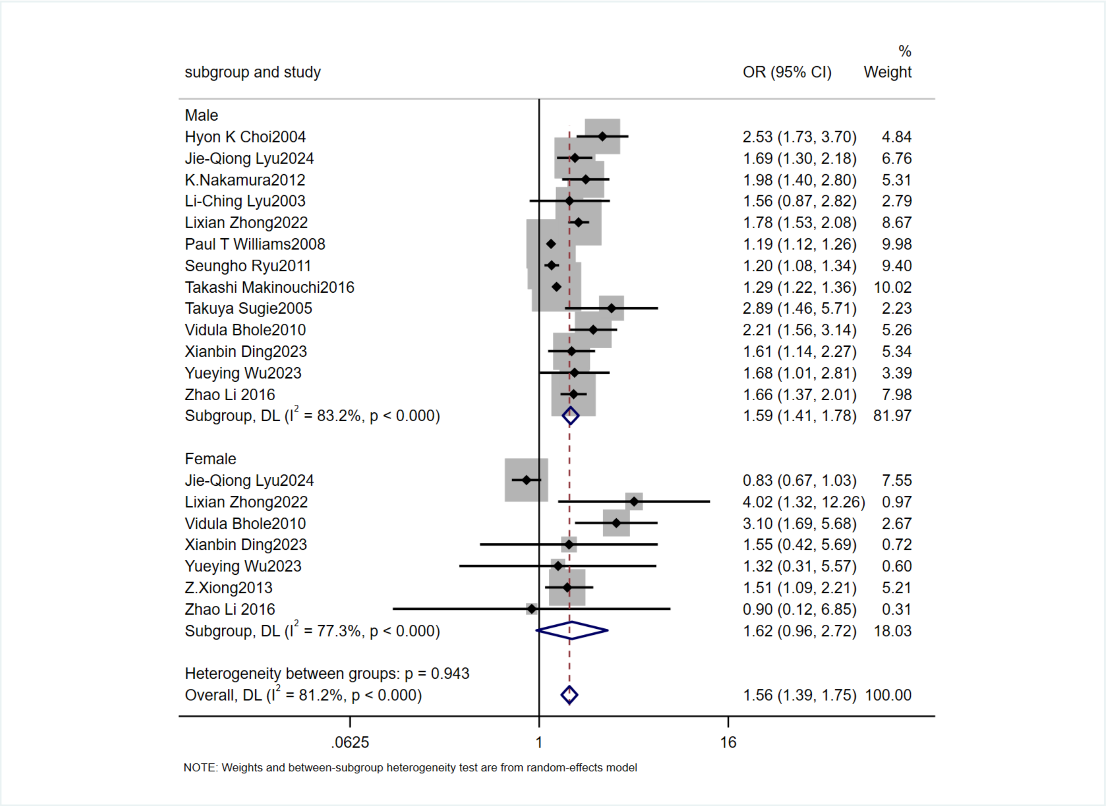

Supplementary Figure 6. Subgroup analysis of the relationship between alcohol.consumption and hyperuricacidemia/gout by gender - Forest Plot.
